# Lipidomic, Transcriptomic, and BSA-660K Single Nucleotide Polymorphisms Profiling Reveal Characteristics of the Cuticular Wax in Wheat

**DOI:** 10.3389/fpls.2021.794878

**Published:** 2021-11-24

**Authors:** Jun Zheng, Chenkang Yang, Xingwei Zheng, Suxian Yan, Fei Qu, Jiajia Zhao, Yanxi Pei

**Affiliations:** ^1^State Key Laboratory of Sustainable Dryland Agriculture, Institute of Wheat Research, Shanxi Agricultural University, Linfen, China; ^2^College of Life Science, Shanxi University, Taiyuan, China

**Keywords:** epicuticular wax, lipidomic, transcriptomic, BSA-660K SNP, lipid metabolism

## Abstract

Plant epidermal wax helps protect plants from adverse environmental conditions, maintains the function of tissues and organs, and ensures normal plant development. However, the constituents of epidermal wax and the regulatory mechanism of their biosynthesis in wheat have not been fully understood. Wheat varieties with different wax content, Jinmai47 and Jinmai84, were selected to comparatively analyze their waxy components and genetic characteristics, using a combination of lipidomic, transcriptomic, and BSA-Wheat 660K chip analysis. Through lipidomic analysis, 1287 lipid molecules were identified representing 31 lipid subclasses. Among these, Diacylglycerols (DG), (O-acyl)-ω-hydroxy fatty acids (OAHFA), wax ester (WE), Triacylglycerols (TG), and Monoradylglycerols (MG) accounted for 96.4% of the total lipids in Jinmai84 and 94.5% in Jinmai47. DG, OAHFA, and WE were higher in Jinmai84 than in Jinmai47 with the content of OAHFA 2.88-fold greater and DG 1.66-fold greater. Transcriptome sequence and bioinformatics analysis revealed 63 differentially expressed genes related to wax biosynthesis. Differentially expressed genes (DEGs) were found to be involved with the OAHFA, DG, and MG of synthesis pathways, which enriched the wax metabolism pathway. Non-glaucous and glaucous bulks from a mapping population were used to identify single nucleotide polymorphisms (SNP) via 660K chip analysis. Two loci centered on chromosomes 2D and 4B were detected and the locus on 4B is likely novel. These data improve understanding of complex lipid metabolism for cuticular wax biosynthesis in wheat and lay the foundation for future detailed investigation of mechanisms regulating wax metabolism.

## Introduction

Cuticular wax is a hydrophobic barrier between the above-ground surface of wheat plants and the surrounding air. It plays a significant role in protecting wheat against both biotic and abiotic stresses. The benefits of cuticular wax include preventing non-stomatal water loss; protection from high temperature and ultraviolet radiation; reducing retention of dust, pollen, and air pollutants on plant surfaces; and enhancing resistance to high salt, drought, low temperature, viruses, bacteria, and pests (Hafiz et al., 2015; Philippe et al., 2020; Arya et al., 2021). Glaucousness has been described as “the visual manifestation of waxiness” by Richards et al. (1986). The cuticular wax content of leaves can be an effective selection criterion in the development of drought-tolerant wheat cultivars (Song et al., 2016; Laskoś et al., 2021). Therefore, a better understanding in wheat of the chemical constituents of cuticular wax, its biosynthesis, and genetic basis would benefit wheat improvement research.

Tulloch and Weenink (1969) used gas-liquid chromatography to analyze the composition of cuticular waxes firstly. Bianchi et al. (1980) studied the cuticular waxes of the ‘Chinese Spring’ and found that the main components were n-alkanes, esters, aldehydes, free alcohols, β-diketones, and hydroxyl-β-diketones. Later new cuticular waxes, such as 2-alkyl alcohols, benzyl alcohols, phenylethyl alcohols, and hydroxyphenyl ethanol were detected on wheat flag leaves and peduncles (Racovita et al., 2016). Recently, Lavergne et al. (2018) found that alkanes (C20-C42), fatty acids (C7-C34), ketones (C9-C35), and primary alcohols (C22-C33) were present in the cuticular wax from the epidermis of wheat leaves and stems. Studies of the chemical constituents of cuticular waxes on wheat leaves mostly used gas chromatography-mass spectrometry (GC-MS) recently. Plant cuticular waxes are known to be organic solvent-extractable complex mixtures of hydrophobic lipids, consisting mostly of very-long-chain fatty acids (VLCFAs) and their derivatives (Xue et al., 2017). Because of the complexity of wax biosynthesis and the limitations of previous detection methods, it is likely there are still many lipid components of cuticular waxes that have not been detected. In addition, the differences between waxy and waxless varieties are not clear at present, which limits the study of the wax biosynthesis pathway. To investigate the pathway of wax biosynthesis in wheat it is critical that the chemical components and content of cuticular waxes are accurately identified.

Hexaploid wheat contains three subgenomes (A, B and D), and all three may contain loci controlling various traits, including glaucousness (Ling et al., 2013). However, previous quantitative trait locus (QTL) studies showed that wax gene loci in common wheat were present primarily on the B and D genomes. It was possible that *Triticum urartu*, the ancestor of A genome, showed green glossy phenotype without β-diketone in the cuticular waxes. Quantitative trait loci (QTLs) for waxes located on different chromosomes have been reported and only loci on 3AL, 2DS, 2B, 3B, and 7B were identified using high-density SNP chips (Börner et al., 2002; Mason et al., 2010; Bennett et al., 2012; Suchismita et al., 2015; Li et al., 2017, 2021a; Mohammed et al., 2021). Although some QTLs and genome segments have been identified for wax-related traits, there are fewer wax-related QTLs and tightly linked markers compared to other traits such as disease-resistance, flowering phase, and yield. Genes involved in wax biosynthesis in wheat have also been reported. *TaFAR5* encoded acyl-CoA reductase in Sinong 2718 and was linked to the biosynthesis of primary alcohols of leaf blade cuticular wax in wheat (Wang Y. et al., 2014). Hen-Avivi et al. (2016) found the wheat *W1* locus contains a mediating β-diketone biosynthesis gene cluster, which encodes proteins of type-III polyketide synthases, hydrolases, and cytochrome P450s related to fatty acid hydroxylases. Ten Fatty acyl-CoA reductase (FAR) genes were found in the diploid grass *Aegilops tauschii*, which were related to the synthesis of primary alcohols C16, C18, C26, C24, and C28 (Wang et al., 2017). *TaCER1-1A* was involved in the biosynthesis of C33 alkanes (Li et al., 2019). Enoyl-CoA reductase *TaECR* is a core enzyme in epidermal wax synthesis of wheat and silencing *TaECR* can reduce epidermal wax and attenuate conidia germination of the powdery mildew pathogen (Kong et al., 2020). β-ketoacyl CoA synthase (KCS) is a key rate-limiting enzyme for the synthesis of VLCFAs. Thirty-three KCS genes representing 12 subgroups in barley were identified by genome-wide analysis. The barley KCS gene family showed different response characteristics under drought stress (Tong et al., 2020). When *TaKCS6* was knocked out in wheat, both cuticular wax accumulation and germination of *Blumeria graminis f. sp. tritici* conidia were impeded on the leaves (Wang et al., 2019). The wax-related genes reported in wheat were obtained primarily by either map base cloning or reverse genetics approaches. Consequently, mapping and cloning of waxy gene/QTL based on the latest research results and developing corresponding molecular markers will be helpful to accelerate stress-resistant genetic breeding programs in wheat.

Since the chemical constitution and genetic basis of cuticular wax from wheat are complex and not fully understood, a combination of transcriptomics, lipidomics, and other multi-omics techniques should be an effective means to study the chemistry of cuticular wax and its genetic basis. In the present study, ultraperformance liquid chromatography tandem mass spectrometry (UPLC-MS/MS) was used to compare glaucous and non-glaucous wheat for the first time, and combined with transcriptome sequencing, to explore further the genetic basis and the chemical composition of epicuticular wax in wheat. Furthermore, we mapped a new wax locus using the 660K SNP chip. These results improve understanding of complex lipid metabolism for cuticular wax biosynthesis and lay the foundation for future detailed investigation of mechanisms regulating wax metabolism.

## Materials and Methods

### Plant Materials and Experimental Design

Jinmai47 is non-glaucous wheat cultivar, which has been used as a control cultivar in dry land areas north of Huang-Huai-Hai River Basin and in Shanxi Province since it was approved in the 1990s. Jinmai84 is glaucous variety approved in 2008.

A population of 230 doubled haploid lines derived from a cross between Jinmai47 and Jinmai84 was generated. The DH population was planted in field under well-watered condition at Linfen (36°48′N, 111°30′E, altitude 450 m) in Shanxi Province in China. Parental lines and DH lines were planted with three replications in a randomized complete block. Each plot consisted of two 1.3 m rows with 19 seeds per row. The DH population and parents were sown in mid-October 2019 and harvested on June 10, 2020. Meanwhile, the well-watered plots were watered with 700 m^3^/hectare at pre-overwintering, jointing and grain filling, respectively.

### Microstructure Observation

To study the microstructure of wheat cuticular wax, scanning electron microscopy (SEM) was performed to observe the wax crystallites deposited on both sides of flag leaf surfaces of glaucous Jinmai84 and non-glaucous Jinmai47. Fresh flag leaves were collected at maturity taking care not to disturb the leaf surface, attached with paper clips to stiff sheets of paper, and dried at 37°C in an oven. Before scanning, dried samples were carefully cut into 5 mm × 5 mm pieces, attached to sample stage, and sputtered with gold powder using the CrC-150 Sputtering System. Images of the leaf surface were captured with an SEM (SJM-6610LV, Japan).

### Analysis of Wax Composition With Ultraperformance Liquid Chromatography Tandem Mass Spectrometry

To extract epicuticular wax, five flag leaves with the same area were taken each from Jinmai84 and Jinmai47 at maturity, placed in 50 mL beakers, chloroform was added until the leaves were soaked, and then the beakers were shaken slowly for 1 min. After the leaves were removed, the beakers were placed in a fuming cupboard to allow the chloroform evaporate to a volume of 0.5–1 ml. The extracts were transferred to glass sample vials and allowed to dry in a fume hood naturally.

Separation was performed on a UPLC Nexera LC-30A. The chromatography column was at 45°C. The flow rate was 300 μL/min. Mobile phase A was acetonitrile water solution (acetonitrile: water = 6:4, V/V) and phase B was acetonitrile isopropanol solution (acetonitrile: isopropanol = 1:9, V/V). The gradient elution was programmed as follows: 0–2 min with 30% B, 2–25 min with 30–100% B, and 25–35 min with 30% B. Samples were placed in a 10°C automatic sampler and analyzed by mass spectrometry with a Q Exactive mass spectrometer. Electrospray ionization (ESI) was performed in positive and negative ion modes. ESI source conditions were as follows: sheath gas flow rate 45 arb, auxiliary gas flow rate 15 arb, collision gas flow rate 1 arb, spray voltage 3.0 kV, capillary temperature 350°C, atomization temperature 300°C, S-Lens RF Level 50%, and MS_1_ scanning range M/Z 200–1,800. The mass charge ratio of lipid molecules and lipid fragments was obtained by collecting 10 fragment maps (MS_2_ scan, HCD) after each full scan. MS_1_ had a resolution of 70,000 at M/Z 200 and MS_2_ had a resolution of 17,500 at M/Z 200.

### RNA-Seq Data Analysis

Fresh flag leaves of the same growth stage from Jinmai84 and Jinmai47 were collected, quickly submerged in liquid nitrogen, packed in dry ice, and sent to Shanghai Applied Protein Technology Company for transcriptomic sequencing.

Gene expression levels were estimated using the RSEM software package. The FPKM (fragments per kilobase per million mapped reads) method was used to calculate the expression levels. An adjusted P value (padj < 0.05) and fold change (FC) ratio (| Log2 FC| ≥ 1) were used to determine the differentially expressed genes (DEGs) between the two varieties using DESeq R package. Gene Ontology (GO) and Kyoto Encyclopedia of Genes and Genomes (KEGG) enrichment analyses of the DEGs were performed to understand the biological significance of the DEGs.

### Bulked-Segregant Analysis

Leaf tissue samples were collected from 30 glaucous and 30 non-glaucous DH lines in the mapping population at the mid-flowering stage. These samples were frozen in liquid nitrogen and stored at −80°C until used for total DNA extraction. Genomic DNA was extracted by the CTAB method. The quality and quantity of the DNA were verified using 1.0% agarose gels and a Nanodrop 2000 spectrophotometer, and then were used to construct two DNA bulks for SNP and marker validation. The Wheat 660K SNP array containing 630,517 SNPs, a high throughput genotyping tool developed by Institute of Crop Sciences Chinese Academy of Agricultural Sciences, was used to test the two bulks at China Golden Marker Biotech Corporation (CGMB, Beijing, China). The frequency distribution of polymorphic SNPs in each chromosome was analyzed using Microsoft Excel 2016^1^. The target chromosomes with potential loci were determined based on the frequency distribution of polymorphic SNPs in each chromosome.

## Results

### Wax Morphology

Flag leaf glaucousness in the two parents was easily distinguishable, with the non-glaucous Jinmai47 having a green glossy phenotype and the glaucous Jinmai84 having a bluish green waxy coating on the flag leaf, sheaths, and spikes (Supplementary Figure 1).

**FIGURE 1:**
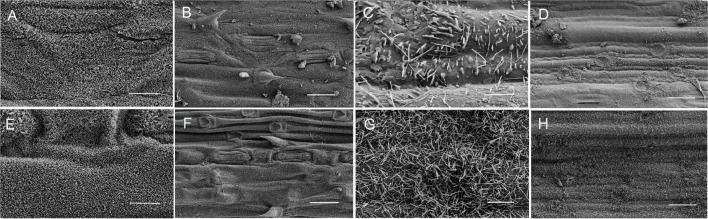
Epicuticular wax crystals on the surfaces of flag leaves from non-glaucous DHs **(A–D)** and glaucous DHs **(E–H)** detected by scanning electron microscope (SEM). **(A,B,E,F)** Adaxial surface of flag leaf. **(C,D,G,H)** Abaxial surface of flag leaf. Panels **(A,C,E,G)** were assessed at 10,000 × magnification, Scale bars = 2 μm; Panels **(B,D,F,H)** were assessed at 2,000 × magnification, Scale bars = 10 μm.

The micromorphology of the epicuticular wax on the adaxial and abaxial surfaces of flag leaves from non-glaucous Jinmai47 and glaucous Jinmai84 was observed by SEM (Figure 1). The glaucous flag leaves showed a dense accumulation on each surface, whereas the wax crystallites deposited surfaces of non-glaucous flag leaves were different. The adaxial surface of both had platelet-shaped crystal structures but the glaucous flag leaves were more densely covered by thick wax crystallites. The content of waxy crystals on the abaxial surface of non-glaucous flag leaves were significantly reduced with both rod-like and platelet-shaped crystals, while waxy crystal on the abaxial surface of none-glaucous flag leaves were mostly rod-like.

### Wax Components

**FIGURE 2:**
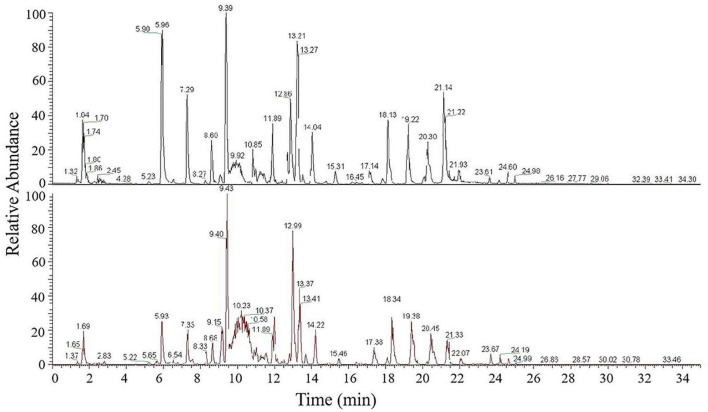
UPLC-MS/MS base peak intensity chromatograms acquired in positive ionization mode of Jinmai47 **(top)** and Jinmai84 **(bottom)**.

To investigate genes involved in wax biosynthesis in wheat, we analyzed the wax composition of flag leaves from non-glaucous Jinmai47 and glaucous Jinmai84. Ultraperformance liquid chromatography-tandem mass spectrometry was used to quantitatively detect the wax composition and content. The signals in positive ionization mode were mainly attributed to 15 subclasses of 972 species of lipids including TG, DG, MG, ceramides (Cer), CerP, monogalactosyl-diacylglycerol (MGDG), digalactosyl-diacylglycerol (DGDG), sulfoquinovosyl-diacylglycerol (SQDG), WE, phosphatidylcholines (PC), phosphatidylethanolamine (PE), phosphatidylglycerol (PG), phosphatidyl inositol (PI), phosphatidylserine (PS) and phosphatidylinositol phosphate (PIP) (Figure 2). Sixteen subclasses of 315 species of lipids consisting of OAHFA, Cer, Cardiolipin (CL), MGDG, DGDG, SQDG, phosphatidic acid (PA), PC, PE, PG, PI, PIP, lysophosphatidyl cholines (LPC), lysophosphatidyl ethanolamine (LPE), lysophosphatidylglycerol (LPG) and sphingomyelin (SM) were detected in negative ionization mode (Supplementary Figure 2). Lipids detected in both positive and negative ionization mode consisted of 1,287 lipid molecules representing 31 lipid subclasses. We identified five lipid classes with fatty acyls, glycerolipids, glycerophospholipids, sphingolipids, and saccharolipids. A total of 748 species of glycerolipids were detected, of which TG was the most frequent with 532 species, DGs the next most common with 191 species, followed by MGs with 25 species. There were 131 species of glycerophospholipids including 19 PCs, 31 PEs, 21 PIs, 33 PGs, three PAs, five PSs, three LPCs, two LPEs, eight CLs, five PIPs, and one LPG. There were 200 species of sphingolipids, including 187 Cers, nine CerPs, and four SMs. There were 88 kinds of SLs, including 41 MGDGs, 22 DGDGs, 25 SQDGs. Furthermore, 63 species of OAHFAs and 57 species of WEs, which belonged to fatty acyls, were recognized (Table 1). Among these lipids categories, the content of DG, OAHFA, WE, TG, and MG were highest, accounting for 96.4 and 94.5% of the total lipids of Jinmai84 and Jinmai47, respectively.

**TABLE 1:** Wax content are means ± SD (mg/g).

**lipid classes**	**Jinmai47 (mg/g)**	**Jinmai84 (mg/g)**	**FC**	**Lipid number**
TG	36.92 ± 3.47	42.35 ± 5.12	1.15	532
DG	312.56 ± 19.23	518.50 ± 21.11**	1.66	191
MG	8.48 ± 1.02	12.22 ± 1.91*	1.44	25
MGDG	3.95 ± 0.73	4.35 ± 0.89	1.11	41
DGDG	6.16 ± 1.68	6.88 ± 1.87	1.12	22
OAHFA	85.61 ± 5.59	246.58 ± 19.35***	2.88	63
WE	49.61 ± 5.99	73.72 ± 11.75**	1.49	57
Cer	7.13 ± 1.61	9.72 ± 1.28*	1.36	196
PI	3.62 ± 0.72	4.24 ± 1.70	1.17	21
Others	7.41 ± 1.20	8.06 ± 2.48	1.08	136
Total	521.47	926.72	1.78	1287

*FC represents Jinmai84/Jinmai47.*

Levels of significance obtained from Student’s *t*-test are indicated as follows: *P < 0.05; **P < 0.01; ***P < 0.001.

The main wax components of Jinmai84 and Jinmai47 were DG, OAHFA, WE, TG and MG and these showed little difference between the two varieties. However, there were significant differences in wax content. The total wax content of Jinmai84 and Jinmai47 were 926.72 and 521.47 mg/g, respectively. Jinmai84 had a thicker waxy layer. The relative contents of the various lipid components of Jinmai84 and Jinmai47 were nearly identical, with both having DG as the highest content accounting for 55.96 and 59.93% of the total lipid content, respectively. Next were OAHFA with 16.41 and 26.61% and WE with 9.51 and 6.96%, respectively. Comparing absolute contents in Jinmai84 versus Jinmai47, OAHFA was 2.88-fold greater, DG was 1.66-fold greater, and DG, OAHFA, WE, Cer and MG (*P* < 0.05) were significantly higher (Table 1).

### Analysis of Differential Lipid Molecules

**FIGURE 3:**
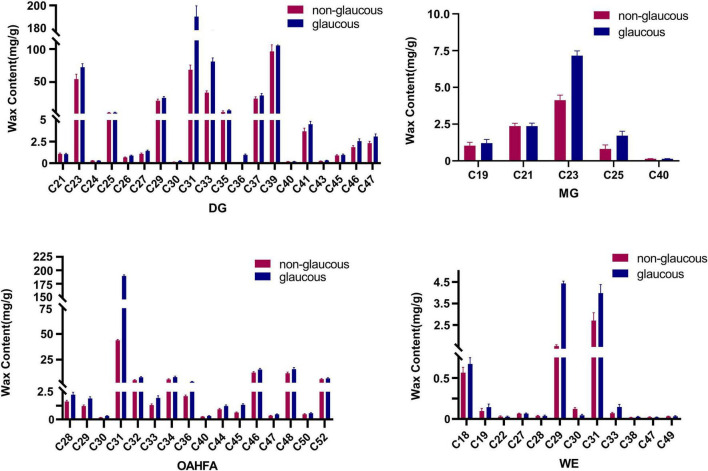
The content of the main lipid molecules in cuticular wax of wheat flag leaves from non-glaucous Jinmai47 and glaucous Jinmai84. Major molecular species composition of DG (Diacylglycerols), MG (Monoradylglycerols), OAHFA [(O-acyl)-ω-hydroxy fatty acids], and WE (Wax ester) determined by UPLC-MS/MS.

In terms of the carbon atom numbers of various lipid molecules in wax of flag leaves in the two parents, the main components were C30-C69 for TG, C31-C39 for DG, and C21-C23 for MG. Chain lengths of C31, C32, C34, C46, and C48 were the main components in OAHFA, and C18, C29, and C31 were most common in WE. Significant differences between the two wheat varieties occurred for lipid molecules C31 DG and C31 OAHFA and the lipid molecules C29 and C30 in WE varied considerably (Figure 3). The content of TG (C30-C69) showed little variation between the wheat varieties.

According to FC > 2, FC < 0.5 and *P*-value < 0.05, 105 lipid molecules with large differences were identified for cluster analysis (Table 2). There were 85 lipid molecules upregulated and 20 downregulated in Jinmai84 compared with Jinmai47 (Supplementary Figure 3 and Supplementary Table 1). Between varieties the lipid molecules with greatest differences within the DG, OAHFA, WE categories were DG (30:4e), OAHFA (15:0/16:0), and WE (13:0/18:3) (Supplementary Figure 4).

**TABLE 2:** Lipid molecules showing significant differences between in two wheat varieties (*P*-value < 0.05, log_2_ (FC) > 2).

**Polarity**	**Lipid**	**M/Z**	**Ion formula**	**Jinmai47 ± SD (μg/g)**	**Jinmai84 ± SD (μg/g)**	**log2(FC)**	***P*-value**
NEG	OAHFA (15:0/16:0)	495.4419	C_31_ H_59_ O_4_	22,755.54 ± 2691.40	105,904.59 ± 19,285.70	2.22	4.83E-03
POS	DG (28:1e)	514.483	C_31_ H_64_ O_4_ N_1_	1724.02 ± 334.26	10,872.80 ± 1741.88	2.66	2.83E-03
POS	DG (28:1e)	497.4564	C_31_ H_61_ O_4_	1616.88 ± 489.20	30848.15 ± 4049.11	4.25	1.74E-03
POS	DG (30:4e)	519.4408	C_33_ H_59_ O_4_	323.91 ± 50.34	11918.08 ± 713.19	5.20	3.68E-04
POS	DG (30:5e)	517.4251	C_33_ H_57_ O_4_	279.17 ± 29.77	3460.19 ± 265.70	3.63	7.51E-03
POS	DG (32:1e)	570.5456	C_35_ H_72_ O_4_ N_1_	69.89 ± 14.76	362.71 ± 51.89	2.38	1.76E-03
POS	DG (27:2e)	481.4251	C_30_ H_57_ O_4_	29.76 ± 7.11	132.54 ± 19.68	2.15	1.52E-03
POS	DG (28:6)	501.3575	C_31_ H_49_ O_5_	15.49 ± 0.56	181.63 ± 34.44	3.55	4.40E-03
POS	WE (17:0/14:2)	480.4775	C_31_ H_62_O_2_ N_1_	1194.16 ± 169.16	5855.60 ± 735.14	2.29	1.67E-03
POS	WE (13:0/18:3)	478.4619	C_31_ H_62_O_2_ N_1_	303.26 ± 36.26	1444.87 ± 203.75	2.25	2.56E-03
POS	WE (13:0/18:3)	461.4353	C_31_H_62_O_2_	99.87 ± 15.42	1078.94 ± 224.96	3.43	5.24E-03
POS	WE (15:1/16:2)	461.4353	C_31_H_57_ O_2_	10.59 ± 2.05	45.40 ± 12.76	2.10	1.91E-02
POS	WE (17:1/14:2)	478.4619	C_31_H_60_ O_2_ N_1_	10.86 ± 1.29	55.36 ± 6.40	2.35	1.51E-03
POS	Cer (d31:1)	478.4619	C_31_ H_60_ O_2_ N_1_	5.53 ± 0.36	27.73 ± 6.48	2.33	8.53E-03
POS	Cer (d30:0)	501.499	C_30_ H_65_ O_3_ N_2_	1.26 ± 0.30	5.79 ± 1.25	2.20	5.54E-03
POS	Cer (d31:1)	496.4724	C_31_ H_62_ O_3_ N_1_	0.12 ± 0.02	20.12 ± 1.65	7.40	7.00E-04
NEG	Cer (t31:1)	556.4583	C_32_ H_62_ O_6_ N_1_	0.07 ± 0.02	0.29 ± 0.04	2.08	9.81E-04
NEG	SM (d44:2)	825.6855	C_48_ H_94_ O_6_ N_2_ P_1_	5.65 ± 0.38	27.16 ± 4.51	2.27	4.29E-03

### RNA-Seq Analysis

**FIGURE 4:**
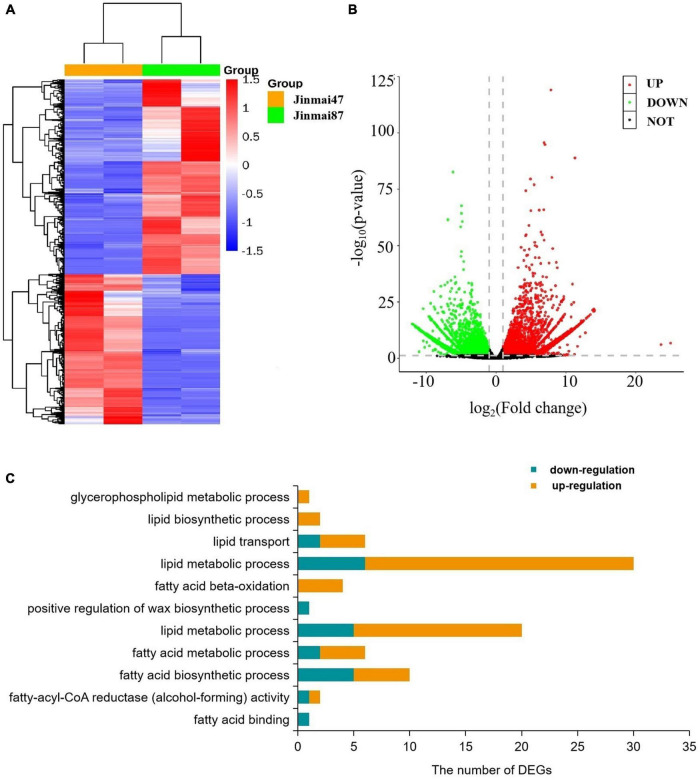
Transcriptome data analysis. **(A)** Heatmap of differentially expressed genes. Each column represents a sample (two biological replicates) and each row represents a gene. **(B)** Volcano plot of differentially expressed genes. **(C)** Gene Ontology (GO) based enrichment analysis (*P*-value ≤0.05) of differentially expressed genes (DEGs) where the *X*-axis represents GO terms and the *Y*-axis indicates the number of DEGs.

To explore the waxy metabolism-related genes in wheat, transcriptome analysis was carried out on both wheat varieties. From 78.17–87.00 million clean reads were generated in each library (Supplementary Figures 5, 6), and from 45.38 to 56.40% were compared to the genome. A total of 120,745 unigenes were sequenced by Blastx alignment against the NCBI non-redundant (NR), Swiss-Prot, KEGG (Supplementary Figure 7), and GO annotations. Among these, 314 genes were related to wax metabolism. In total, 9438 genes (DEGs) showed some degree of differential expression (Figures 4A,B). A total of 63 DEGs were involved in wax metabolism, with 17 genes upregulated and 46 genes downregulated in the non-glaucous Jinmai47 compared to glaucous Jinmai84. KEGG enrichment analysis showed DEGs related to wax metabolism enriched in following pathways: 15 for glycerophospholipid metabolism, four for fatty acid degradation, six for steroid biosynthesis, four for ABC transporters, three for biosynthesis of unsaturated fatty acids, two for cutin, suberine, and wax biosynthesis, four for fatty acid biosynthesis, seven for fatty acid elongation, seven for fatty acid metabolism, seven for glycerolipid metabolism, and three for sphingolipid metabolism. In addition, we found six transcription factors related to wax transport and two transcription factors related to the regulation of wax biosynthesis. Based on GO annotation, DEGs were mainly enriched in biological process and molecular function. For molecular function, one related to fatty acid binding and two genes were enriched in fatty-acyl-CoA reductase (alcohol-forming) activity. For biological process, there were 10 genes related to fatty acid biosynthetic process, six genes related to fatty acid metabolic process, 25 related to lipid metabolic process, one related to positive regulation of wax biosynthetic process, four genes related to fatty acid beta-oxidation, 30 related to lipid metabolic process, six related to lipid transport, two related to lipid biosynthetic process, and one related to glycerophospholipid metabolic process (Figure 4C).

**FIGURE 5:**
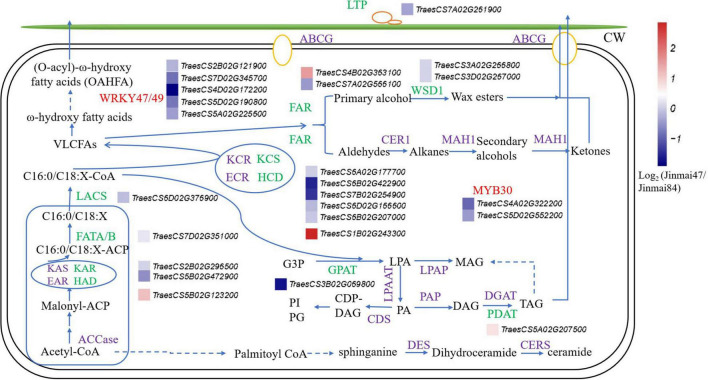
The pathway of epicuticular wax biosynthesis in wheat. The green represents genes detected via transcriptomic analysis, purple represents genes not detected, and the red represents transcription factors. Relative expression profiles (blue-white-red intensity scale) of unigenes are shown. Red indicates upregulated expression and blue indicates downregulated expression. Log2 (Jinmai47/Jinmai84) represents the gene expression fold change, where Jinmai47/Jinmai84 refers to the ratio of FPKM reads.

To identify the genes involved in the wax synthesis pathway, the wax synthesis pathway was established from previous reports and the genes labeled with their corresponding expression levels (Yasuhiro et al., 2015; Zhang et al., 2020; Li et al., 2021b; Figure 5). The results combined the lipidomic and transcriptomic analyses and confirmed that most of the genes related to wax synthesis were significantly downregulated in Jinmai47 compared with Jinmai84. DEGs involved in the synthesis pathways of OAHFA, DG, and MG were also identified. In the process of VLCFAs biosynthesis, genes encoding β-Ketoacyl-ACP reductase (KAR), fatty acyl-ACP thioesterase (FATA/B), long-chain acyl-CoA synthase (LACS), and KCS were downregulated. Hydroxyacyl-ACP dehydratase (HAD) genes (a member of fatty acid synthase (FAS) family) and HCD (β-hydroxyacyl CoA dehydratase) genes (a member of fatty acid elongase (FAE) family) were upregulated in Jinmai47, presumably due to the negative feedback of the C18/C16-ACP and C18/C16 acyl-CoAs decrease. VLCFAs can be used to generate primary alcohols and wax esters via the acyl reduction pathway, and produce n-alkanes, aldehydes, secondary alcohols and ketones via the decarboxylation pathway. Also, VLCFAs are hydroxylated to form ω-hydroxy VLCFA, which participate in the formation of OAHFA. The expression of FAR and WSD1 genes in the acyl reduction pathway were significantly downregulated, which explained why the content of WE detected in lipidomic analysis was significantly lower in Jinmai47. DGAT is the key rate-limiting enzyme for TG synthesis, but its expression level did not change significantly, whereas the PDAT expression level was slightly upregulated. Correspondingly, there was no change in content of TG identified in the two wheat varieties. Moreover, the expression of LTP1 related to wax transport and MYB30, a transcription factor regulating wax synthesis, were both significantly downregulated.

### Gene Location by 660K Single Nucleotide Polymorphisms

**FIGURE 6:**
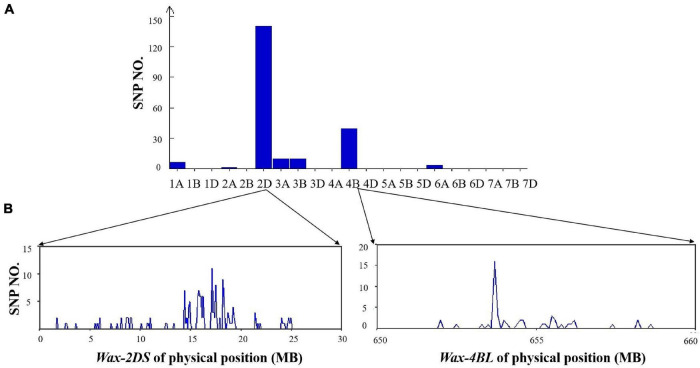
**(A)** Distribution of polymorphic SNPs in each chromosome identified by the 660K SNP array. **(B)** Physical position of SNPs in chromosome 2D and 4B based on 660K SNP physical map.

From the 660K SNP chip results, there were 217 polymorphic SNPs in the mixed pool of the two DH lines, 140 of which were distributed on 2DS (64.5%) and 49 on 4BL (22.6%), indicating that *Wax-2DS* and *Wax-4BL* were related to wax synthesis (Figure 6).

The number of SNPs per 0.1Mb was counted. *Wax-2DS* was mapped through 2D markers in a 14–19Mb genomic region containing 127 genes. Compared with *GLOSSY1* reported previously, the physical position of *Wax-2DS* overlapped with SSR 4 and SSR 6 (12.4–19.6 Mb), which were used for fine localization (Li et al., 2021a). Thus, *GLOSSY1* and *Wax-2DS* are the same locus and our result obtained with BSA-660K localization was accurate. The wax locus *Wax-4B* is likely novel and was mapped to an approximately 25 Mb genomic interval on chromosome 4BL of wheat, which contained 142 genes and lies at 640–665 Mb based on the International Wheat Genome Sequencing Consortium (IWGSC) reference genome for the wheat cultivar Chinese Spring.

Transcriptomic sequencing results were used to identify the differentially expressed genes in the two regions, and 11 differentially expressed genes were found in the 14–19Mb of 2DS as follows: *TraesCS2D02G039400, TraesCS2D02G039900*, *TraesCS2D02G040900*, *TraesCS2D02G041600*, *TraesCS2D02G041700*, *TraesCS2D02G043500*, *TraesCS2D02G044100*, *TraesCS2D02G044600*, *TraesCS2D02G047500*, *TraesCS2D02G049000*, and *TraesCS2D02G050700*. Seven differentially expressed genes were identified in 4BL: *TraesCS4B02G366900, TraesCS4B02G363700, TraesCS4B02G363100, TraesCS4B02G365300, TraesCS4B02G365600, TraesCS4 B02G363200*, and *TraesCS4B02G367500.* We performed the annotation of gene function for these DEGs (Supplementary Table 2). Among them, *TraesCS4B02G363100* was identified as fatty-acyl-CoA reductase, which catalyzed the chemical reactions and pathways involving lipid; *TraesCS4B02G363700* was involved in lipid metabolic process.

## Discussion

### Analysis of Wax Composition

The glaucous and non-glaucous phenotypes, which can be quickly identified and recorded in the field, are major traits for drought adaptation and have been widely used for breeding of drought-resistant, high-yield wheat varieties (Xue et al., 2017). The glaucous phenotype is caused by epicuticular wax deposition, which affects non-stomatal transpiration and regulates gas exchange between plant and environment together with stomata. When stomata are closed, epicuticular wax can reduce leaf water loss and CO_2_ exchange under drought stress. However, glaucous wheat does not always show drought tolerance and many non-glaucous varieties are also adapted to drought-prone environments (Bi et al., 2017; Willick et al., 2018). Drought resistance of wheat is a complex and comprehensive trait. The mechanisms of drought resistance including drought escape, avoidance and tolerance strategies, are affected by the interaction and regulation of multiple genetic factors (Snježana et al., 2008). Present researches have shown that epidermal wax can prevent leaves from water loss under drought conditions. For example, glaucous wheat should be preferred for drought-resistant varieties in near-isogenic lines and high-generation inbred lines (Nizam and Marshall, 1986; Richards et al., 1986; Tsunewaki and Ebana, 1999). Moreover, the effect of different wax components on drought resistance is expected to be clarified with further research. Panikashvili et al. (2007) found that the decrease of plant drought resistance was caused by the reduction of keratin and wax, especially C29 alkane. Therefore, a fuller understanding of the composition and regulation mechanism of epicuticular wax in wheat might enable a more sophisticated manipulation of glaucousness in breeding drought-tolerant wheat varieties. The cuticular waxes of the Chinese Spring wheat were composed of n-alkanes, esters, aldehydes, free alcohols, β-diketone, and hydroxyl-β-diketone (Bianchi et al., 1980). On wheat flag leaves and peduncles, 2-alkyl alcohols, benzyl alcohols, phenylethyl alcohols, and hydroxyphenyl ethanol were detected successively (Racovita et al., 2016). Recently, researchers identified that alkanes (C20-C42), fatty acids (C7-C34), ketones (C9-C35), and primary alcohols (C22-C33) were in the cuticular wax from wheat leaves and stems (Lavergne et al., 2018). As it turns out, the accurate analysis of wax components is closely related to the development of detection equipment.

As a high-throughput analysis technique, lipidomics has been widely used recently. UPLC-MS/MS, which has the advantages of high sensitivity and good resolution, is the most efficient detection method. The lipidomics of six microalgae were analyzed by UPLC-MS/MS, and 102 species of triglycerides were identified (Karen et al., 2011). Lu et al. (2019) detected a total of 13 lipid subclasses and 218 species of triglycerides in different tissues of rape. Yan et al. (2021) identified 525 lipids in walnut and the main molecules were TG (18:2/18:2/18:3) and DG (18:2/18:2). In rice during different storage periods, lipidomic analysis revealed 21 lipid subclasses and 277 lipid molecules of which FA, OAHFA, DG, and TG were discovered for the first time (Zhang et al., 2021). The present study is the first to use UPLC-MS/MS to analyze the composition of epidermal wax of wheat flag leaves and identify the main components of epidermal wax, including DG(C21-47), OAHFA(C31-52), WE(C18-33), TG(C30-69), and MG(C19-40). The contents of these five lipid subclasses were the highest in epidermal waxes, among which DG, OAHFA, and WE showed the greatest differences between wheat varieties. In addition, these lipid molecules were found in wheat epidermal wax for the first time. The ω-hydroxy fatty acids (ω-FAHFA) are a type of VLCFA, also known as OAHFAs, and are biological surfactants in tear membranes, skin, and sebum (Wood Paul, 2020) and is also present in rice (Zhang et al., 2021). The present study is the first report of this lipid molecule in wheat epidermal wax and its function deserves further study. WE are produced by acylation of fatty alcohols, and the content of WE (C29-31) increase significantly between the two varieties. It is interesting that there were 20 downregulated lipid molecules in Jinmai84, which consisted of C16 and C18 fatty acids. Because the KCS and KCR genes were upregulated and C29 WE and C31 OAHFA were higher in Jinmai84, more C16 and C18 fatty acids may be used to form VLCFAs, which participate in the formation of WE and OAHFA.

### Analysis of the Wax Synthesis Pathway

The biosynthesis of wax has been characterized in rice, and the metabolic, transport and accumulation mechanism of the epicuticular wax have been clarified (Lee and Suh, 2013, 2015). Wax biosynthesis mainly occurs in epidermal cells (Li-Beisson et al., 2013). In VLCFA biosynthesis, one of the key reactions is the activation of fatty acid acyl chains to fatty acyl-CoA. Previous studies have indicated that LACS1/CER8 and LACS2 located on the endoplasmic reticulum were involved in wax synthesis (Lü et al., 2009). Transcriptome results in the present study showed that the LACS gene was downregulated in Jinmai47 compared with Jinmai84. Ultra-long chain fatty acids (C20-C34) were synthesized by fatty acid elongase (FAE), a multienzyme complex. The process involves four enzymatic reactions, including KCS, β-ketoacyl CoA reductase (KCR), HCD, and enoyl-CoA reductase (ECR). Among them, KCS was the key rate-limiting enzyme, and its downregulation was why the content of C31 DG, C31 OAHFA and C29 WE differed significantly between wheat varieties. The final reaction forming TG, DG, and MG is catalyzed by glycerol-3-phosphate acyltransferase (GPAT). *GPAT1-8* genes are important for the synthesis of extracellular lipid polymeric esters in *Arabidopsis* (Li-Beission et al., 2009). *GPAT4-8* contributes to cuticle synthesis. These enzymes showed greater catalytic efficiency for terminal hydroxyl or carboxyl fatty acids than fatty acids which have not been taken, indicating that they function after oxidation reactions (Li et al., 2007). The MYB transcription factor family member, which regulated the biosynthesis of plant epicuticular wax, is mainly related to drought and water stress. *MYB30* can regulate the expression of the *KCS1/2*, *PAS2/HCD*, *CER3*, and *LTPG1* genes, and participates in the lipid biosynthesis and VLCFAs extension pathways, inducing the accumulation of VLCFAs (Raffaele et al., 2008; Zhang et al., 2012). The epicuticular wax on the leaf surface significantly increased in rice overexpressing *WRKY89* and decreased in RNAi-mediated *WRKY89* suppression lines (Wang et al., 2007). The role of the *WRKY47/49* transcription factor in wheat is unclear, but its expression levels were significantly downregulated in the present study in Jinmai47. Some genes were identified as key genes, and these candidate genes provide new targets for further genetic modification.

The content and chemical composition of epidermal wax in various plant species differ. Even in the same species it varies with growth stage, tissue, and organs (Kunst and Samuel, 2003; Kosma et al., 2009; Bernard and Joubès, 2013). In the present study, the wax composition of wheat was shown to differ from other plants and the synthesis and regulation of waxes in wheat was explored. By combining lipidomic and transcriptomic analyses, it was shown that OAHFA was formed by the hydroxylation of VLCFAs. *CYP86A2, CYP86A4, CYP86A7* and *CYP86A8* mutants in *Arabidopsis thaliana* show abnormal structure and changes in composition of cuticle, suggesting that these genes are closely related to the hydroxylation of ω chains VLCFAs in cuticle biosynthesis (Fich et al., 2016). OAHFA differed significantly in two parents in lipidomic. However, in our transcriptomic data, *TraesCS7B02G209300* was related to the hydroxylation of ω chains VLCFAs, but there was no difference in expression level between wheat varieties.

### 660K Single Nucleotide Polymorphisms Analysis

Epicuticular waxes in leaf and stem are mainly controlled by two groups of alleles, the glaucous loci (*W1* and *W2*) and the non-glaucous loci (*Iw1* and *Iw2*). *W1* and *Iw1* are closely linked on chromosome 2BS. The distance between *W2* and *Iw2* was great, with *W2* at the proximal and *Iw2* at the distal end of 2DS (Tsunewaki, 1966). *Iw3*, a wax inhibitor locus which was closely linked with *XPSP3000*-*XWL3096*, is located on chromosome 1BS (Tsunewaki and Ebana, 1999; Adamski et al., 2013; Wu et al., 2013). *Iw3* was found in durum wheat (*Turgidum subsp*) and inhibited production of β-diketones and primary alcohols in epicuticular wax of glume (Wang J. et al., 2014). *W3* was involved in β-diketones synthesis on chromosome 2BS in a novel wax mutant in wheat cultivar Bobwhite (Zhang et al., 2015). *W4* on 3DL was found in the natural population of *Aegilops tauschii* (Nishijima et al., 2018). Semi-dominant gene *W5* was mapped in the 194kb region of 7DL in a wax-deficient mutant from Jimai22 and transcriptomic analysis showed that genes related epicuticular wax were significantly downregulated (Li et al., 2020, 2021b). Through fine mapping using bulked-segregant analysis (BSA) analysis and SSR markers, *W1* was mapped to a 0.93 cM interval of chromosome 2BS (Lu et al., 2015). In addition, Li et al. (2017) used SNPs to obtain a major QTL *QFlg. Hwwgr-3al*, which was located in a 4.4cM interval of chromosome 3AL. The *Glossy1* gene in a Jimai 22 mutant was mapped to a 308.1kbp region of 2DS chromosome using SSR markers (Li et al., 2021a). Recently genome-wide association analysis was used to analyze the genotypes of 1106 wheat varieties with the glaucous phenotype in flag leaves, and two major QTLs on 3A and 2B were identified (Würschum et al., 2020).

There have been relatively few reports on the mapping and cloning of waxy QTL/genes in wheat. In the present research, two main loci located on 2D and 4B were identified using the 660K high density chip in non-glaucous and glaucous bulks. The locus on 2D was consistent with previous studies (Li et al., 2021b), while the locus on 4B appears to be a novel locus. Furthermore, we got candidate genes involved in wax biosynthesis in wheat leaf surface and identified a DEG on 4B belonging to fatty acyl-CoA reductases (FARs). In previous research, *TaFAR5* was mapped to wheat chromosome 4DS (Wang Y. et al., 2014). In future studies, fine-scale linkage mapping of the new locus should be conducted to provide new genetic resources related to wax synthesis. In addition to identifying candidate genes for wax accumulation, the present study revealed the chemical structure of the epicuticular wax in flag leaves of wheat. In combination with lipidomic and transcriptomic analyses, these findings provide a theoretical foundation for the in-depth exploration of biological function of the wax synthesis genes of wheat and may further the genetic analysis of related species.

## Data Availability Statement

The data presented in the study are deposited in the NCBI SRA repository, accession number PRJNA772972. The SRA records will be accessible with the following link after the indicated release date 2022.01.01: https://www.ncbi.nlm.nih.gov/sra/PRJNA772972.

## Author Contributions

JZhe and FQ designed the experiments. CY and SY performed the experiments. CY and JZha analyzed the data. SY and XZ drafted the manuscript. JZhe and YP revised the manuscript. All authors contributed to the article and approved the submitted version.

## Conflict of Interest

The authors declare that the research was conducted in the absence of any commercial or financial relationships that could be construed as a potential conflict of interest.

## Publisher’s Note

All claims expressed in this article are solely those of the authors and do not necessarily represent those of their affiliated organizations, or those of the publisher, the editors and the reviewers. Any product that may be evaluated in this article, or claim that may be made by its manufacturer, is not guaranteed or endorsed by the publisher.

## Footnotes

1
https://www.microsoft.com/zh-cn/
