# Supplementary material for: Lipidomic, Transcriptomic, and BSA-660K Single Nucleotide Polymorphisms Profiling Reveal Characteristics of the Cuticular Wax in Wheat
Source: Front Plant Sci. 2021 Nov 24;12:794878. doi: 10.3389/fpls.2021.794878 (PMC8652291; doi:10.3389/fpls.2021.794878)
Supplement: Supplementary file 1 [file Data_Sheet_1.docx]

Supplementary Material


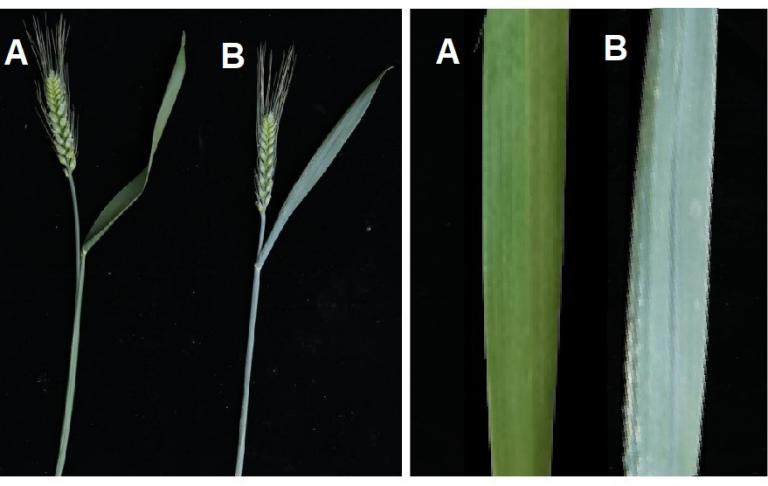


Figure S1. The phenotype of Jinmai47 (non-glaucous) for A and Jinmai84 (glaucous) for B


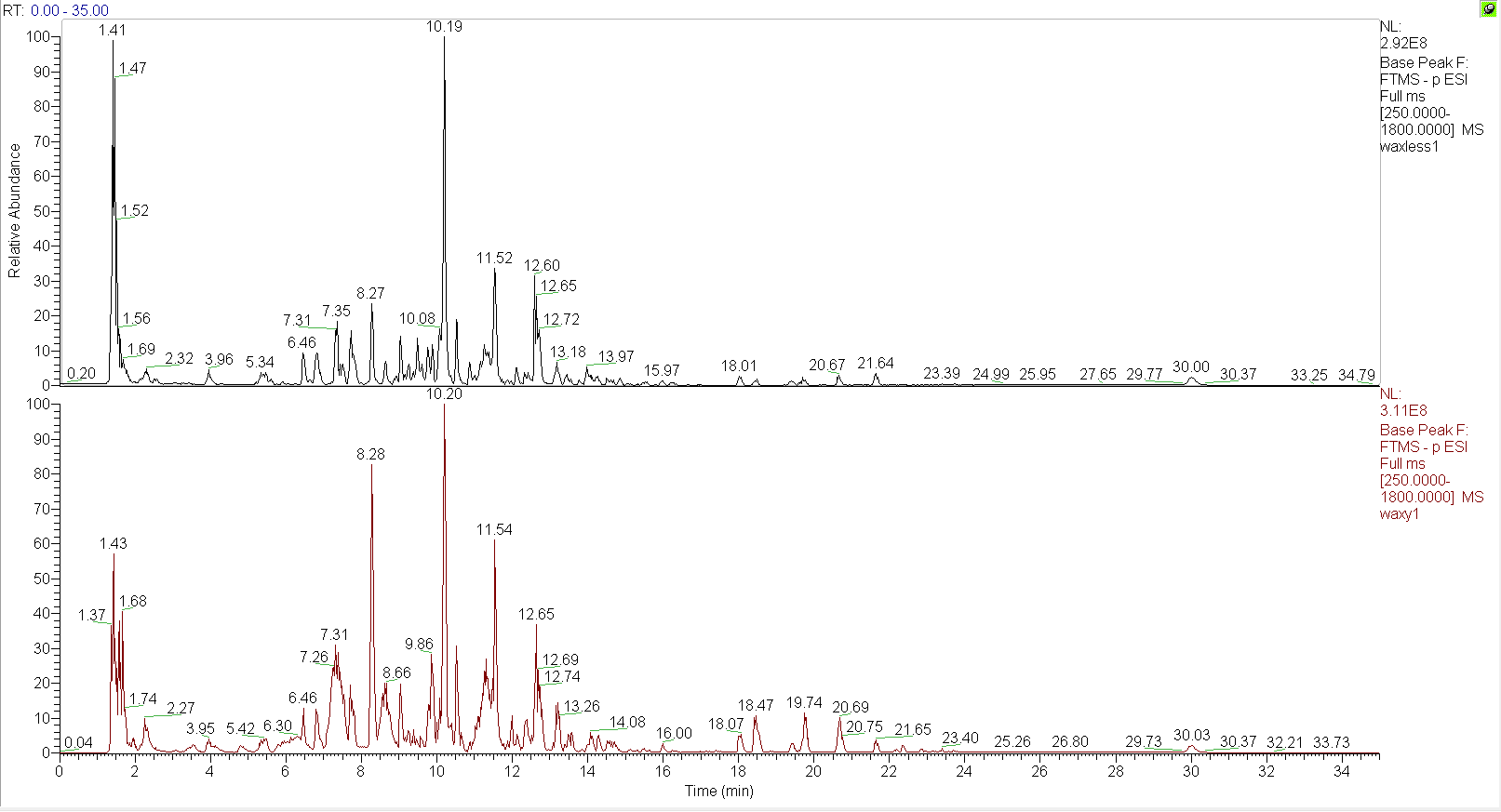


Figure S2 UPLC-MS/MS base peak intensity chromatograms acquired in negative ionization mode of Jinmai47 and Jinmai84. The spectra from top to bottom are Jinmai47 (negative), and Jinmai84 (negative).


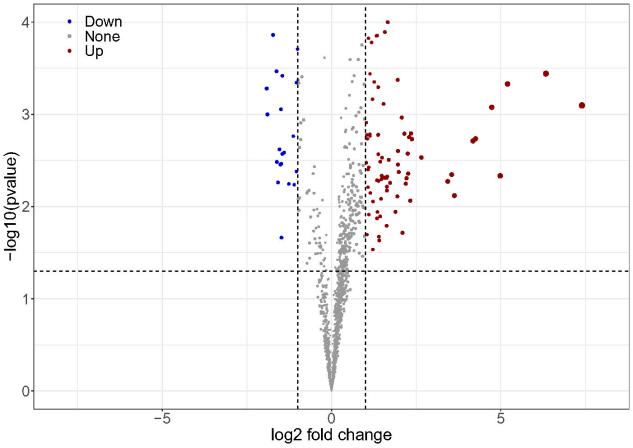


Figure S3 Volcano plot of differentially lipids. The dots represent lipid molecules, among which the blue and red dots are differential lipid molecules that satisfies FC<0.5, FC>2 and P value<0.05.


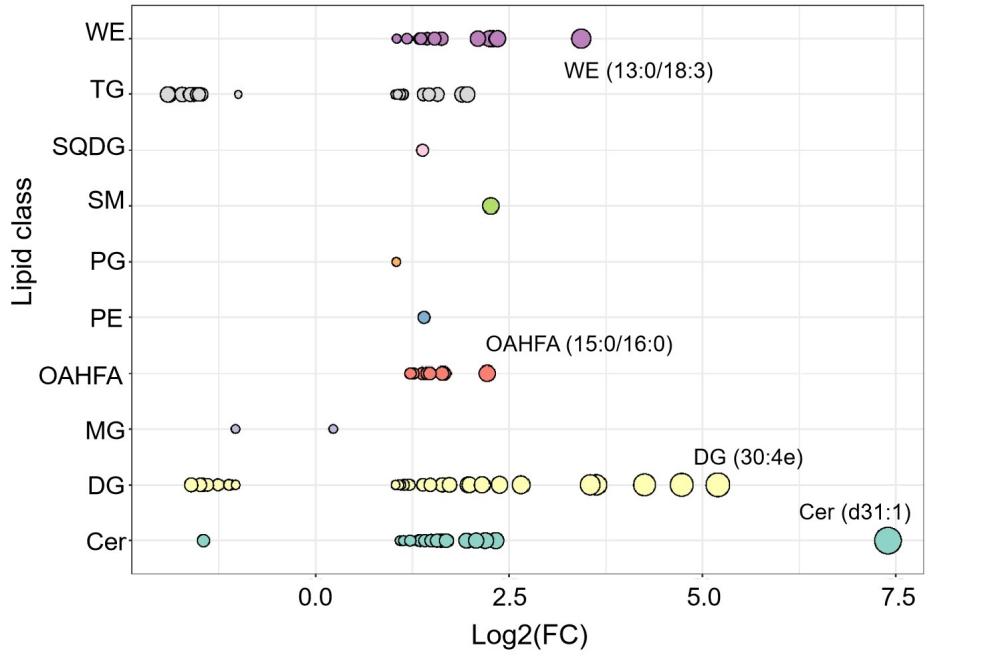


Figure S4 Lipid molecules in cuticular wax of flag leaves showing significant differences between wheat varieties Jinmai47 and Jinmai84. X-axis represents log2 (Jinmai84/Jianmai47), Y-axis indicates lipid subclasses.


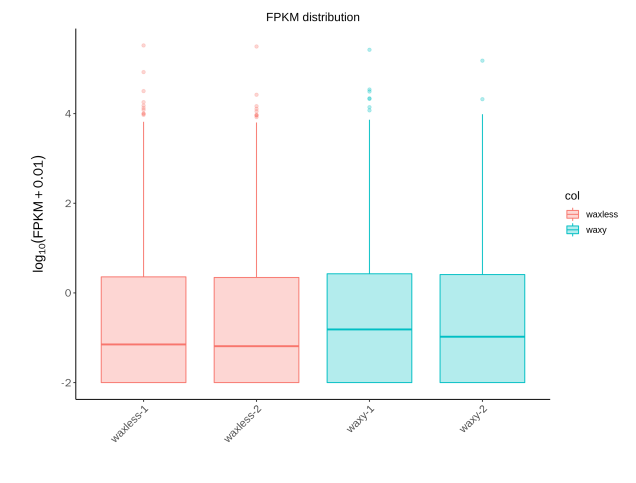


Figure S5 Boxplot graph of FPKM in Jinmai47 (waxless) and Jinmai84 (waxy). X-axis represents the sample name; Y-axis represents Log_10_ (FPKM+0.01), indicating the distribution range of gene expression level.


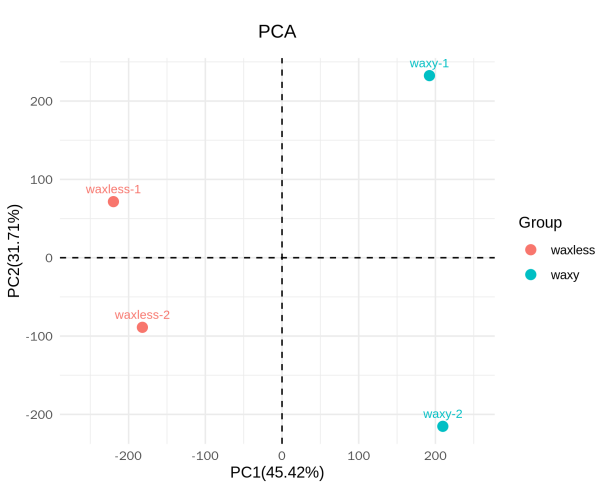


Figure S6 [Principal](C:/Users/35701/AppData/Local/youdao/dict/Application/8.9.9.0/resultui/html/index.html#/javascript:;) [component](C:/Users/35701/AppData/Local/youdao/dict/Application/8.9.9.0/resultui/html/index.html#/javascript:;) [analysis](C:/Users/35701/AppData/Local/youdao/dict/Application/8.9.9.0/resultui/html/index.html#/javascript:;) (PAC) in transcriptomic in Jinmai47 (waxless) and Jinmai84 (waxy). The two biological replicates were set up for each group.


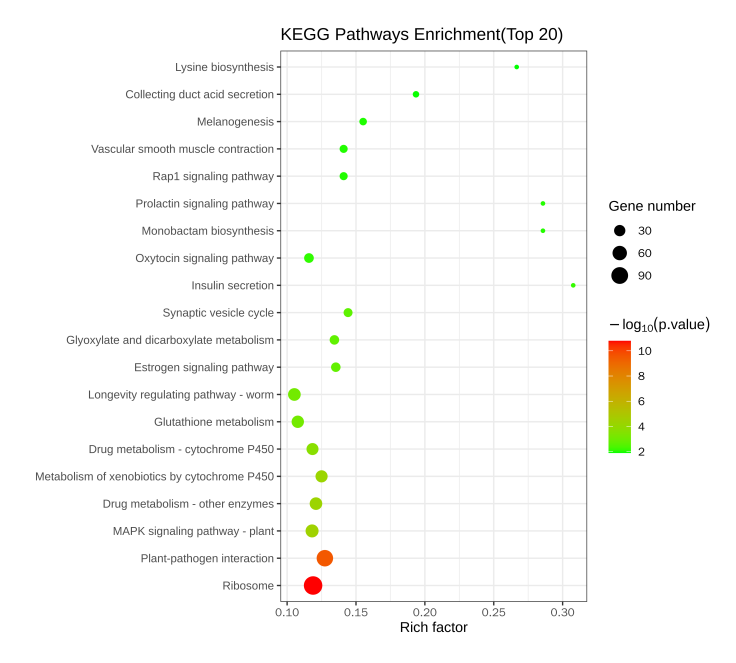


Figure S7 KEGG pathways enrichment of DEGs. X-axis represents *P*-value; Y-axis represents the name of pathway. The size of rich factor was represented by the color of dots, and the larger the value was, the closer the color was to red. The number of DEGs contained under each pathway was represented by the size of scattered dots.
